# Hypoxylonol F Isolated from *Annulohypoxylon annulatum* Improves Insulin Secretion by Regulating Pancreatic β-cell Metabolism

**DOI:** 10.3390/biom9080335

**Published:** 2019-08-02

**Authors:** Dahae Lee, Buyng Su Hwang, Pilju Choi, Taejung Kim, Youngseok Kim, Bong Geun Song, Noriko Yamabe, Gwi Seo Hwang, Ki Sung Kang, Jungyeob Ham

**Affiliations:** 1 School of Pharmacy, Sungkyunkwan University, Suwon 16419, Korea; 2 College of Korean Medicine, Gachon University, Seongnam 13120, Korea; 3 Freshwater Bioresources Utilization Bureau, Nakdonggang National Institute of Biological Resources, Sangju 37242, Korea; 4 Natural Products Research Institute, Korea Institute of Science and Technology, Gangneung 25451, Korea; 5 Division of Bio-Medical Science and Technology, University of Science and Technology, Daejeon 34114, Korea

**Keywords:** *Annulohypoxylon annulatum*, insulin, PI3K, Akt, PPARγ, PDX-1

## Abstract

Insulin plays a key role in glucose homeostasis and is hence used to treat hyperglycemia, the main characteristic of diabetes mellitus. *Annulohypoxylon annulatum* is an inedible ball-shaped wood-rotting fungus, and hypoxylon F is one of the major compounds of *A. annulatum*. The aim of this study is to evaluate the effects of hypoxylonol F isolated from *A. annulatum* on insulin secretion in INS-1 pancreatic β-cells and demonstrate the molecular mechanisms involved. Glucose-stimulated insulin secretion (GSIS) values were evaluated using a rat insulin ELISA kit. Moreover, the expression of proteins related to pancreatic β-cell metabolism and insulin secretion was evaluated using Western blotting. Hypoxylonol F isolated from *A. annulatum* was found to significantly enhance glucose-stimulated insulin secretion without inducing cytotoxicity. Additionally, hypoxylonol F enhanced insulin receptor substrate-2 (IRS-2) levels and activated the phosphatidylinositol 3-kinase/protein kinase B (PI3K/Akt) pathway. Interestingly, it also modulated the expression of peroxisome proliferator-activated receptor γ (PPARγ) and pancreatic and duodenal homeobox 1 (PDX-1). Our findings showed that *A. annulatum* and its bioactive compounds are capable of improving insulin secretion by pancreatic β-cells. This suggests that *A. annulatum* can be used as a therapeutic agent to treat diabetes.

## 1. Introduction

Diabetes is a common chronic metabolic disease that affects millions of people worldwide and is closely related to modern lifestyle [1]. Hyperglycemia is the hallmark of diabetes and is caused by impaired insulin synthesis or secretion [2]. Pancreatic β-cells located in the islet of Langerhans play a critical role in insulin synthesis and secretion, which controls energy metabolism [3]. Typically, the elevation in blood glucose levels after a meal stimulates insulin secretion [4]. However, when insulin secretion is inadequate to meet the metabolic demand, blood glucose levels remain high, resulting in diabetes [5,6].

The expression of the insulin receptor (IR) in pancreatic β-cells plays an essential role in β-cell development and function during diabetes progression. Overexpression of the IR leads to increased β-cell proliferation and insulin production [7]. The IR can activate its main substrates, insulin receptor substrate-1 (IRS-1) and insulin receptor substrate-2 (IRS-2), followed by the activation of the phosphatidylinositol 3-kinase/protein kinase B (PI3K/Akt) pathway, which is important for regulating pancreatic β-cell function and survival [8]. Activated PI3K/Akt subsequently induces the phosphorylation of transcription factor forkhead box protein O1 (FOXO1), indicating the inhibition of FOXO1 activity. Additionally, the activation of FOXO1 suppresses the expression of peroxisome proliferator-activated receptor γ (PPARγ) and nuclear translocation of pancreatic and duodenal homeobox 1 (PDX-1). Overexpression of PPARγ increases the nuclear translocation of PDX-1 [9,10,11].

Studies have suggested that either the inhibition of FOXO1 or induction of PPARγ mediates the increase in the nuclear translocation of PDX1, stimulating insulin secretion. Therefore, evaluating these pathways will provide novel evidence to support the improvement in insulin secretion.

Although many efforts have been made to develop drugs to prevent and regulate diabetes for several decades, its prevalence has been increasing annually [12]. The commonly used antidiabetic drugs include synthetic insulin and drugs that replace insulin or improve insulin secretion. However, some of these drugs have adverse effects [13]. Metformin is known to induce gastrointestinal side effects, and hypoglycemia and body weight gain are a major concern when using sulfonylureas [4]. Pioglitazone causes an increased risk of bladder cancer [14], cardiovascular events [15,16], skeletal fracture in postmenopausal women [17], weight gain [18], and peripheral edema [19,20]. Therefore, identifying and developing antidiabetic drugs without adverse effects are very important. Natural compounds with insulin-like activity may be favorable alternatives for treating diabetes [21].

To date, active components including dietary fibers, polysaccharides, and other compounds extracted from cultured mycelium, fruiting bodies of medicinal mushrooms, have been reported to exhibit anti-hyperglycemic activity. In our search for the natural compounds of wild mushrooms that improve insulin secretion, we found *Annulohypoxylon annulatum*, and its isolated secondary metabolites, can be a possible candidate to treat diabetes; it has been reported to inhibit tumor angiogenesis. Treatment with hypoxylonols D and E, novel benzo[*j*]fluoranthene derivatives, isolated from *Annulohypoxylon annulatum*, inhibits proliferation of the human umbilical vein endothelial cell line (HUVEC) and human umbilical artery endothelial cell line (HUAEC) as the essential step in tumor angiogenesis [22]. In addition, it has been reported to protect against cisplatin-induced cytotoxicity in the porcine renal proximal tubule epithelial cell line (LLC-PK1) through anti-apoptosis by inhibiting p38, c-Jun N-terminal kinase (JNK), and extracellular signal-regulated kinase (ERK) phosphorylation and caspase-3 cleavage [23].

Here, we report the isolation of hypoxylonol C (**1**), hypoxylonol F (**2**), and 4,5,4′,5′-tetrahydroxy-1,1′-binaphthyl (BNT (**3**)) from *A. annulatum*, along with their activities that improve insulin secretion. This study also elucidated the action mechanism of hypoxylonol F on insulin signaling pathways in INS-1 cells, as hypoxylonol F enhances the phosphorylation of IRS-2 levels, which are associated with the activation of the PI3K/Akt pathway and PDX-1. We found that PPARγ activation plays a major role in the insulin-secreting effect of hypoxylonol F.

## 2. Materials and Methods

### 2.1. General Experimental Procedures

Preparative HPLC was carried out on a LC-Forte/R (YMC Co., Tokyo, Japan) with an ultraviolet (UV) detector (YMC Co., Tokyo, Japan) (230 nm) using a Phenomenex Kinetex C18 column (250 × 21.2 mm, 10 µm, Phenomenex, Torrance, CA, USA), whereas the semi-preparative LC system (Gilson Inc, Middleton, WI, USA) was equipped with a refractive index (RI) detector and a Phenomenex Gemini C6-ph column (250 × 10 mm, 5 µm, Phenomenex, Torrance, CA, USA). NMR spectra were recorded on a Bruker AVACE III 400 spectrometer (Bruker, Billerica, MA, USA) (400 and 100 MHz for ^1^H and ^13^C, respectively) in acetone-*d*_6_. Chemical shifts in the proton and carbon spectra measured in acetone-*d*_6_ were reported in reference to residual solvent peaks at 2.05 and 29.9 ppm, respectively. Ultra-high-performance liquid chromatography (UPLC) ESI mass spectrometry was performed on a Shimadzu LCMS-2020 system (Shimadzu, Kyoto, Japan). High-resolution mass spectra were acquired using a JEOL JMS-700 mass spectrometer (JEOL Ltd, Tokyo, Japan) under electron impact or fast atom bombardment (FAB) conditions at the Korea Basic Science Institute.

### 2.2. Fungal Material

Mushrooms collected from Yeongok-myeon, Gangneung city, Korea, were identified as *A. annulatum*. A voucher specimen (MCO-NP-I-0026) was deposited at the Library of Natural Products Research Institute, Korea Institute of Science and Technology. The genus *Annulohypoxylon* is a member of the Xylariaceae family that has brown to dark brown and phaseoliform single-cell ascospores with a conspicuous full germ slit. Samples from dead wood were washed thoroughly to eliminate extraneous material, lyophilized, and stored in a refrigerator at −15 °C until use.

### 2.3. Extraction and Isolation

The lyophilized *A. annulatum* (280 g) was extracted twice with methanol (3 L) at room temperature and filtered. The methanolic extract (*ca*. 35 g) was suspended in water and then successively partitioned with normal hexane (Hex), ethyl acetate (EA), and normal butanol (BuOH), yielding 5.4, 7.1, and 8.2 g of residue, respectively. To identify active ingredients responsible for insulin secretion, each fraction was evaluated for glucose-stimulated insulin secretion (GSIS) using a rat insulin ELISA kit. The active fraction, the EA-soluble fraction, was first separated by reversed-phase HPLC (Phenomenex C18 column, 250 × 21.2 mm, 10 µm) eluting water (A) and MeCN (B), both containing 0.1% formic acid, at a flow rate of 20 mL/min, using gradient solvent systems (50% B over 5 min, 50–100% B over 50 min, 100% B over 5 min) with a 230 nm UV detector to yield three sub-fractions (A–C). Further purification of each sub-fraction (A, B, and C) was carried out using semi-preparative HPLC with a column fitted with a Phenomenex column (C6-ph, 250 × 10 mm, 5 µm) and RI detector and eluted with 25% aqueous MeOH, at a flow rate of 4 mL/min, to afford pure compounds **1** (*t_R_* 12.9 min, 682 mg, purity 96.4%), **2** (*t_R_* 13.8 min, 407 mg, purity 95.3%), and **3** (*t_R_* 16.0 min, 588 mg, purity 94.5%).

**Figure 1:**
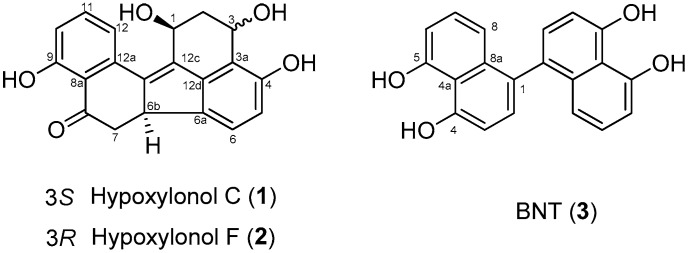
Chemical structures of isolated hypoxylonol C (**1**), F (**2**), and 4,5,4′,5′-tetrahydroxy-1,1′-binaphthyl (BNT) (**3**).

The three compounds (Figure 1) were identified as hypoxylonol C (**1**), hypoxylonol F (**2**), and 4,5,4′,5′-tetrahydroxy-1,1′-binaphthyl (BNT (**3**)) by comparing the subsequent NMR analysis with information in published reports [22,24]. The purity of these compounds was determined by HPLC (Supplementary Materials).

### 2.4. Cell Culture

The INS-1 rat insulin-secreting β-cell line was purchased from Biohermes (Shanghai, China) and cultured in a RPMI-1640 medium (Cellgro, Manassas, VA, USA), supplemented with 1% penicillin/streptomycin (Invitrogen Co., Grand Island, NY, USA), 10% FBS, 11 mM d-glucose, 2 mM l-glutamine, 10 mM HEPES, 0.05 mM 2-mercaptoethanol, and 1 mM sodium pyruvate at 37 °C in a humidified atmosphere with 5% CO_2_.

### 2.5. Cell Viability Assay

Cell viability was evaluated using the Ez-Cytox cell viability detection kit (Daeil Lab Service Co., Seoul, Korea) [25]. INS-1 cells were seeded (1 × 10^4^ cells/well) in 96-well plates. After incubation for 24 h, the cells were treated with test compounds for 24 h and then incubated with Ez-Cytox reagent (10 μL/well) for 2 h. Following incubation, absorbance values were measured at 450 nm using a PowerWave XS microplate reader (Bio-Tek Instruments, Winooski, VT, USA). The cell viability of the control (untreated cells) was regarded as 100%.

The human hepatocellular carcinoma cell lines HepG2 and Hep3B, human cervical carcinoma cell line HeLa, human breast carcinoma cell lines MCF7 and MDA-MB-231, and human glioblastoma cell line T98G were purchased from the American Type Culture Collection (ATCC). The cells were routinely grown in DMEM (Gibco) and RPMI1640 (Gibco), supplemented with 10% fetal bovine serum (Gibco), 100 U/mL penicillin, and 100 µg/mL streptomycin at 37 °C in a humidified atmosphere with 5% CO_2_.

### 2.6. Insulin Secretion Assay

Glucose-stimulated insulin secretion (GSIS) was evaluated using a rat insulin ELISA kit (Gentaur, Shibayagi Co. Ltd., Gunma, Shibukaw, Japan). INS-1 cells were seeded (5 × 10^5^ cells/well) in 12-well plates. After incubation for 24 h, each well was washed twice with Krebs-Ringer bicarbonate HEPES buffer (KRBB, 4.8 mM KCl, 129 mM NaCl, 1.2 mM KH_2_PO_4_, 1.2 mM MgSO_4_, 2.5 mM CaCl_2_, 10 mM HEPES, 5 mM NaHCO3, and 0.1% BSA, pH 7.4) and 2.8 mM glucose. Before treatment, the cells were allowed to starve in fresh KRBB. After incubation for 2 h, the cells were treated with KRBB containing test samples and gliclazide (positive control), and then KRBB containing basal (2.8 mM) and stimulating (16.7 mM) glucose concentrations was added to each well. After incubation for 1 h, the supernatants from each well were collected and centrifuged at 12,000 rpm and 4 °C for 10 min and then GSIS was assessed using a rat insulin ELISA kit according to the manufacturer’s instructions. The glucose stimulation index (GSI) was calculated by dividing the insulin level at the stimulating (16.7 mM) glucose concentration by the insulin level at the basal (2.8 mM) glucose concentration and was compared with the control (untreated glucose-stimulated cells).

### 2.7. Western Blot Analysis

The expression of proteins related to pancreatic β-cell metabolism was evaluated using Western blot analysis [26]. INS-1 cells were seeded (4 × 10^5^ cells/well) in 6-well plates. After incubation for 24 h, the cells were treated with test samples for 24 h and then lysed with RIPA buffer (Cell Signaling, Danvers, MA, USA) containing 1 mM phenylmethylsulfonyl fluoride on ice at 4 °C. Cell lysates were collected and centrifuged at 6000 rpm for 2 min at 4 °C. The supernatants were collected and the concentration of each protein was determined using the Pierce™ BCA protein assay kit (Thermo Scientific, Carlsbad, CA, USA).

The proteins mixed with 4x loading buffer (20 μg/lane) were separated using 10% sodium dodecyl sulfate polyacrylamide gel and transferred to polyvinylidene difluoride membranes, which were further incubated for 1 h with primary antibodies against PPARγ, P-IRS-2, phospho-IRS2 (P-IRS-2), phospho-PI3K (P-PI3K), phosphor-Akt (P-Akt) (Ser473), Akt, PDX-1, and GAPDH (Cell Signaling, Danvers, MA, USA) at room temperature, and incubated again for 1 h with horseradish peroxidase (HRP)-conjugated anti-rabbit secondary antibodies (Cell Signaling, Boston, MA, USA) at room temperature [27]. The proteins (ECL Advance, GE Healthcare, UK) were visualized using a chemiluminescence system (FUSION Solo, PEQLAB Biotechnologie GmbH, Erlangen, Germany) according to the manufacturer’s instructions. The Western blot was repeated three times and representative data presented.

### 2.8. Statistical Analysis

Statistical significance was determined using the One-Way Analysis of Variance (ANOVA) and multiple comparisons with a Bonferroni correction. *p* values of less than 0.05 indicated statistical significance. All analyses were performed using SPSS Statistics ver. 19.0 (SPSS Inc., Chicago, IL, USA).

## 3. Results

### 3.1. Effect of Compounds ***1**–**3*** Isolated from A. annulatum on Glucose-Stimulated Insulin Secretion

**Figure 2:**
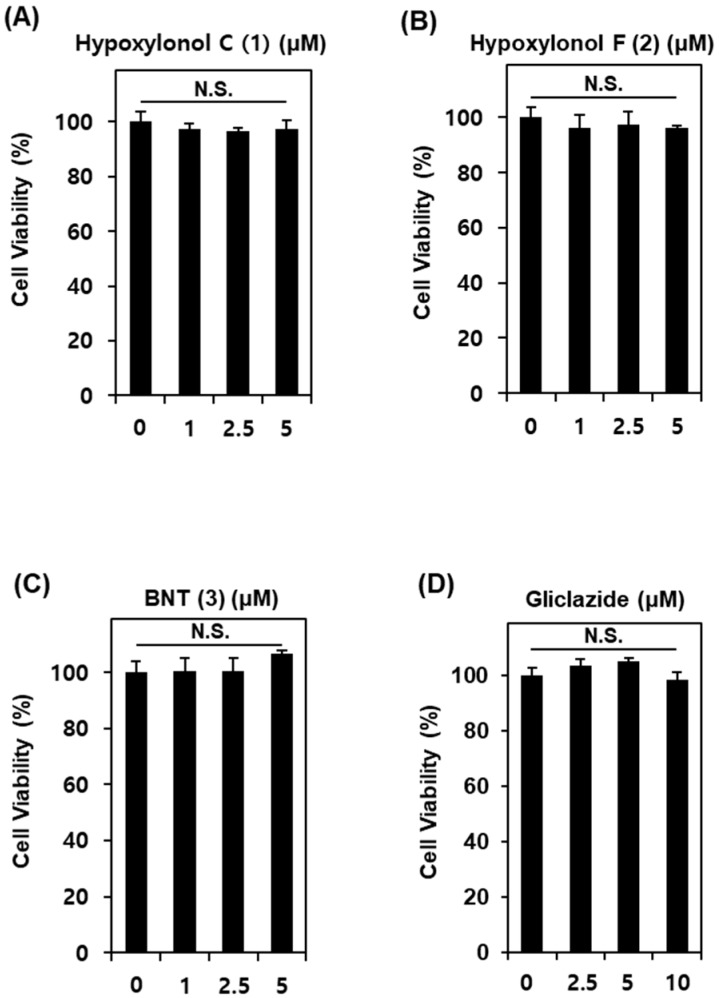
Effects of compounds **1**–**3** isolated from *A. annulatum* on viability of INS-1 cells. Effect of (**A**) hypoxylonol C (**1**), (**B**) hypoxylonol F (**2**), (**C**) BNT (**3**), and (**D**) gliclazide (positive control) when compared with the control (0 μg/mL) on viability of INS-1 cells for 24 h by Ez-Cytox cell viability assay. N.S., not significant: *p* > 0.05 compared with control (0 μM).

**Figure 3:**
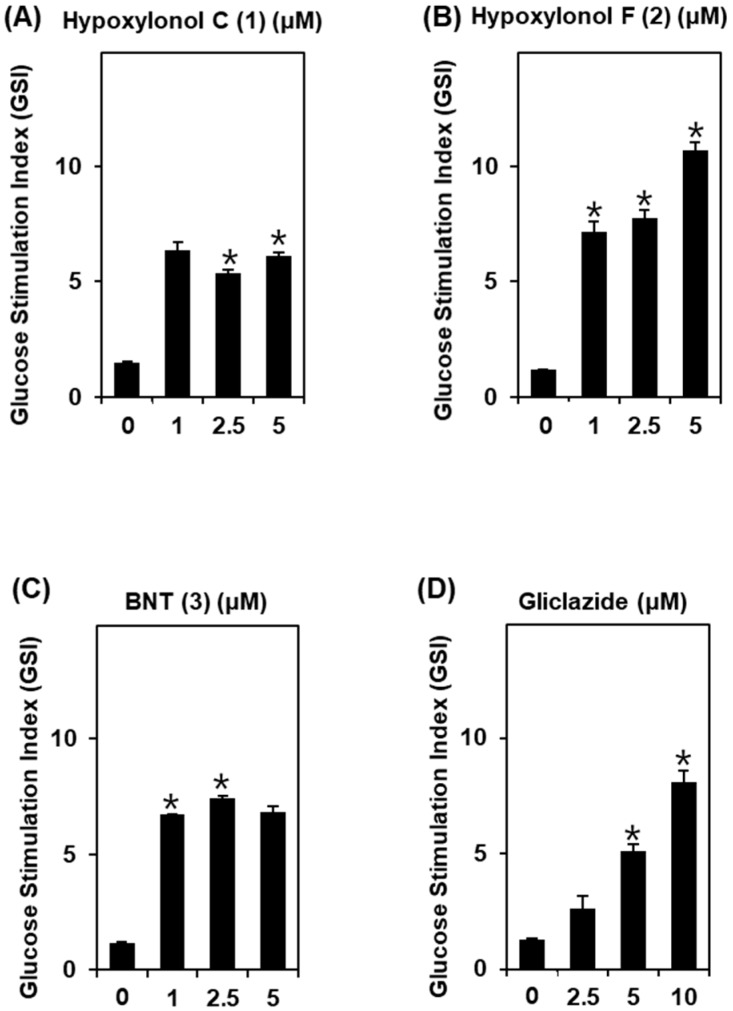
Effect of compounds **1**–**3** isolated from *A. annulatum* on glucose-stimulated insulin secretion in INS-1 cells. Effect of (**A**) hypoxylonol C (**1**), (**B**) hypoxylonol F (**2**), (**C**) BNT (**3**), and (**D**) gliclazide (positive control) on glucose-stimulated insulin secretion in INS-1 cells for 1 h by insulin secretion assay. * *p* < 0.05 compared with control (0 μM).

To determine the non-toxic dose ranges of compounds **1**–**3**, we assessed the cytotoxic effect of various concentrations of compounds **1**–**3** on INS-1 cells. As shown in Figure 2, compounds **1**–**3** at 1, 2.5, and 5 μM show no toxic effects. Additionally, as shown in Figure 3, compounds **1**–**3** lead to an increase in GSI even at 1 μM. The GSI levels are 6.1 ± 0.1, 10.6 ± 0.3, and 6.8 ± 0.2 for compounds **1**–**3** at 5 μM, respectively. Among the three compounds, hypoxylonol F (**2**) leads to the strongest increase in GSI in a dose-dependent manner (Figure 3B).

Based on insulin secretion assays, hypoxylonol F (**2**) from *A. annulatum* stimulates insulin secretion in INS-1 cells without inducing cytotoxicity. Therefore, further mechanistic studies were carried out using hypoxylonol F (**2**).

### 3.2. Effect of Hypoxylonol F (***2***) on the Protein Expression of PPARγ, P-IRS-2, IRS-2, P-PI3K, PI3K, P-Akt (Ser473), Akt, and PDX-1

To investigate the underlying molecular mechanisms by which hypoxylonol F (**2**) affects insulin secretion, Western blotting was performed to quantify the expression of proteins involved in pancreatic β-cell metabolism

**Figure 4:**
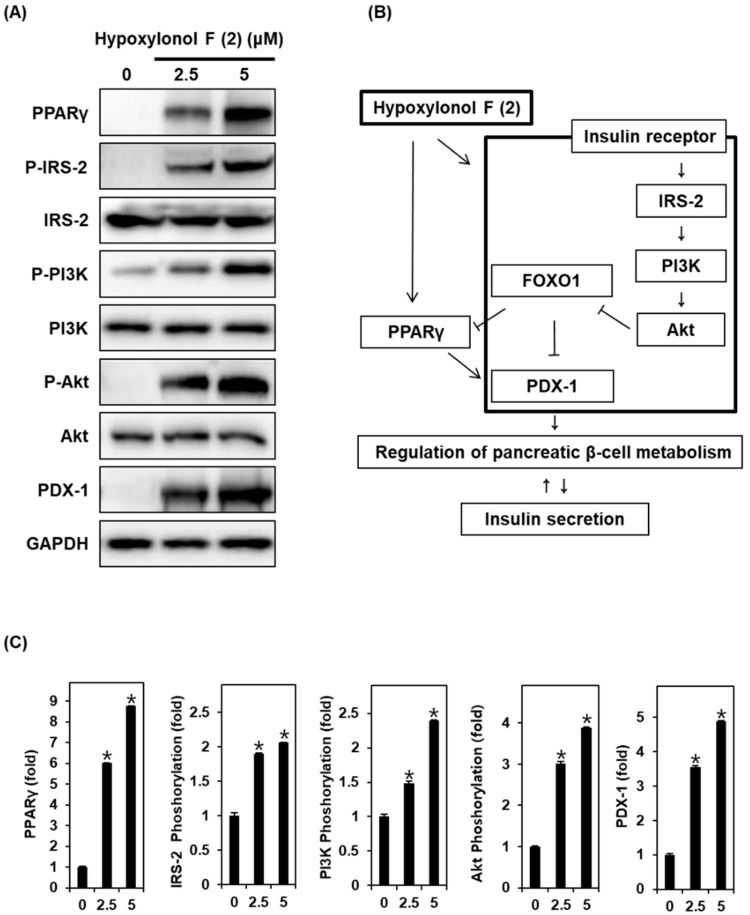
Effect of hypoxylonol F (**2**) on the protein expression levels of peroxisome proliferator-activated receptor γ (PPARγ), P-IRS-2, insulin receptor substrate-2 (IRS-2), P-PI3K, PI3K, P-Akt (Ser473), Akt, and pancreatic and duodenal homeobox 1 (PDX-1) in INS-1 cells. (**A**) Protein expression levels of PPARγ, P-IRS-2, IRS-2, P-PI3K, PI3K, P-Akt (Ser473), Akt, PDX-1, and GAPDH in INS-1 cells treated or untreated with 2.5 μM and 5 μM hypoxylonol F (**2**) for 24 h. (**B**) Schematic illustration of effects of hypoxylonol F (**2**) on the protein expression levels of PPARγ, P-IRS-2, IRS-2, P-PI3K, PI3K, P-Akt (Ser473), Akt, and PDX-1 in INS-1 cells. (**C**) Each bar graphs presents the densitometric quantification of Western blot bands. * *p* < 0.05 compared with control (0 μM).

As shown in Figure 4A, the protein expression levels of PPARγ, P-IRS-2, P-PI3K, P-Akt (Ser473), and PDX-1 are markedly increased by treatment with 2.5 and 5 μM hypoxylonol F (**2**). The bar graphs show the ratio of PPARγ, P-IRS-2, P-PI3K, P-Akt (Ser473), and PDX-1 expression normalized by GAPDH (Figure 4C).

## 4. Discussion

In the present study, we investigated the insulin secretory effects of *A. annulatum* and its bioactive compounds and the underlying mechanisms to find a favorable alternative therapy for diabetes.

To determine insulin secretory function, we assessed the effects of *A. annulatum* and its fractions on GSIS in INS-1 cells. The MeOH extract, EA fraction, and water fraction led to an increase in GSIS in a dose-dependent manner in INS-1 cells without inducing cytotoxicity. Compounds **1**–**3** (hypoxylonol C (**1**), hypoxylonol F (**2**), and BNT (**3**)) isolated from *A. annulatum* also led to an increase in GSIS in a dose-dependent manner in INS-1 cells without inducing cytotoxicity. In response to high blood glucose concentrations, insulin secretion may be influenced by various factors related to insulin synthesis, insulin secretion from secretory granules, and pancreatic β-cell metabolism [28,29]. IRS2-PI3K-Akt signaling plays a key role in pancreatic β-cell metabolism influencing insulin synthesis [30]. The IR and its main intracellular tyrosine kinase substrates, insulin receptor substrate IRS-1 and IRS-2, are involved in growth, function, and insulin secretion in pancreatic β-cells [8].

Previous studies have shown that IR-null mice exhibit glucose tolerance and loss of pancreatic β-cell mass [31,32]. IRS-1 and IRS-2 trigger PI3K, which activates Akt (Ser473) through phosphorylation. The PI3K/Akt pathway plays an important role in islet mass and pancreatic β-cell proliferation by regulating apoptosis, differentiation, and proliferation [33,34]. Previous studies have also shown that the targeted IRS-2 knockout mice leads to β-cell failure [10]. Targeted knockout of the Akt gene in mice resulted in hyperglycemia [35,36]. In contrast, overexpression of the Akt gene resulted in an increase in islet mass and pancreatic β-cell proliferation in mice [33]. Treatment with PI3K inhibitors could reverse insulin secretion with glucose stimulation [37]. In line with previous studies [31,32,33,34,35,36,37], treatment with hypoxylonol F (**2**) downregulated the expression of IRS-2 and its downstream targets, PI3K/Akt, in INS-1 cells.

Some studies have also indicated that phosphorylation of the PI3K/Akt pathway inactivates FOXO1-dependent gene expression. FOXO1 leads to the inhibition of pancreatic β-cell growth and acts as a transcriptional brake, leading to transcriptional restraint in the expression of PPARγ and PDX-1 [4,10]. In contrast, overexpression of PPARγ increases the nuclear translocation of PDX-1 [9,10,11,38]. PPARγ is a known regulator of glucose and lipid homeostasis, inflammation, and cellular proliferation and differentiation [9]. PDX-1, a transcription factor, plays an important role in normal pancreatic development, insulin secretion, and β-cell survival and function. Previous studies have suggested that the FOXO1/PPARγ-mediated pathway plays a crucial role in pancreatic β-cell survival and function in diabetic rats [38]. In line with previous studies, hypoxylonol F (**2**) caused an increase in PPARγ and PDX-1 protein expression.

Consequently, our findings revealed that *A. annulatum* and its bioactive compounds are capable of improving insulin secretion by regulating IRS-2, PI3K, Akt (Ser473), PPARγ, and PDX-1, which are indispensable for maintaining the normal function of pancreatic β-cells. Our study suggests that *A. annulatum* and its compounds can prevent or delay the development of diabetes.

## 5. Conclusions

In conclusion, this in vitro study provided the first knowledge that hypoxylonol F (**2**) isolated from *A. annulatum* significantly promoted GSIS without toxicity in pancreatic β-cells. Our study suggested that IRS-2, PI3K, Akt, PPARγ, and PDX-1 played a major role in this effect. Although further studies are needed to elucidate the molecular mechanism by which hypoxylonol F (**2**) promotes GSIS, there is potential to use hypoxylonol F (**2**) as a naturally occurring antidiabetic agent.

## Supplementary Materials

The following are available online, Scheme S1: Fractionation schemes of *Annulohypoxylon annulatum*, Figure S1: HPLC chromatograms of *Annulohypoxylon annulatum* extract (A), Hypoxylonol C (B), Hypoxylonol F (C), BNT (D), Figure S2: ^1^H NMR NMR spectrum of hypoxylonol C (1), Figure S3: ^13^C-NMR NMR spectrum of hypoxylonol C (1), Figure S4: HRMS of hypoxylonol C (1), Figure S5: ^1^H-NMR NMR spectrum of hypoxylonol F (2), Figure S6: ^13^C-NMR NMR spectrum of hypoxylonol F (2), Figure S7: HRMS of hypoxylonol F (2), Figure S8: ^1^H NMR NMR spectrum of BNT (3), Figure S9: ^13^C-NMR NMR spectrum of BNT (3), Figure S10: HRMS of BNT (3), Figure S11: Effect of hypoxylonol F (2) on the protein expression levels of PPARγ, P-IRS-2, IRS-2, P-PI3K, PI3K, P-Akt (Ser473), Akt, and PDX-1 in INS-1 cells.

## References

[1] Shi B.-Y. (2016). The importance and strategy of diabetes prevention. Chronic. Dis. Transl. Med..

[2] Zhou L. (2015). Investigation of pancreatic β-cell insulin receptor regulation of β-cell growth, function, and survival via a temporal conditional knockout. Electron. Thesis Diss. Repos..

[3] Lamontagne J., Al-Mass A., Nolan C.J., Corkey B.E., Madiraju S.M., Joly E., Prentki M. (2017). Identification of the signals for glucose-induced insulin secretion in INS1 (832/13) β-cells using metformin-induced metabolic deceleration as a model. J. Biol. Chem..

[4] Valeron P.F., de Pablos-Velasco P.L. (2013). Limitations of insulin-dependent drugs in the treatment of type 2 diabetes mellitus. Med. Clin..

[5] Medina M.C., Souza L.C., Caperuto L.C., Anhê G.F., Amanso A.M., Teixeira V.P., Bordin S., Carpinelli Â.R., Britto L.R., Barbieri R.L. (2006). Dehydroepiandrosterone increases β-cell mass and improves the glucose-induced insulin secretion by pancreatic islets from aged rats. FEBS Lett..

[6] Yao X.G., Chen F., Li P., Quan L., Chen J., Yu L., Ding H., Li C., Chen L., Gao Z. (2013). Natural product vindoline stimulates insulin secretion and efficiently ameliorates glucose homeostasis in diabetic murine models. J. Ethnopharmacol..

[7] Torii S., Kubota C., Saito N., Kawano A., Hou N., Kobayashi M., Torii R., Hosaka M., Kitamura T., Takeuchi T. (2018). The pseudophosphatase phogrin enables glucose-stimulated insulin signaling in pancreatic β cells. J. Biol. Chem..

[8] Fu Z., Gilbert E.R., Liu D. (2013). Regulation of insulin synthesis and secretion and pancreatic Beta-cell dysfunction in diabetes. Curr. Diabetes Rev..

[9] Kim H.-S., Hwang Y.-C., Koo S.-H., Park K.S., Lee M.-S., Kim K.-W., Lee M.-K. (2013). PPAR-γ activation increases insulin secretion through the up-regulation of the free fatty acid receptor GPR40 in pancreatic β-cells. PLoS ONE.

[10] Kitamura T., Nakae J., Kitamura Y., Kido Y., Biggs W.H., Wright C.V., White M.F., Arden K.C., Accili D. (2002). The forkhead transcription factor Foxo1 links insulin signaling to Pdx1 regulation of pancreatic beta cell growth. J. Clin. Invest..

[11] Zhang T., Kim D.H., Xiao X., Lee S., Gong Z., Muzumdar R., Calabuig-Navarro V., Yamauchi J., Harashima H., Wang R. (2016). FoxO1 plays an important role in regulating β-cell compensation for insulin resistance in male mice. Endocrinology.

[12] Cordero-Herrera I., Martin M.A., Bravo L., Goya L., Ramos S. (2013). Cocoa flavonoids improve insulin signalling and modulate glucose production via AKT and AMPK in HepG2 cells. Mol. Nutr. Food Res..

[13] Stein S.A., Lamos E.M., Davis S.N. (2013). A review of the efficacy and safety of oral antidiabetic drugs. Expert Opin. Drug Saf..

[14] He S., Tang Y.H., Zhao G., Yang X., Wang D., Zhang Y. (2014). Pioglitazone prescription increases risk of bladder cancer in patients with type 2 diabetes: An updated meta-analysis. Tumour Biol..

[15] Lincoff A.M., Wolski K., Nicholls S.J., Nissen S.E. (2007). Pioglitazone and risk of cardiovascular events in patients with type 2 diabetes mellitus: A meta-analysis of randomized trials. JAMA..

[16] Mizushige K., Tsuji T., Noma T. (2002). Pioglitazone: Cardiovascular effects in prediabetic patients. Cardiovasc. Drug Rev..

[17] Bone H.G., Lindsay R., McClung M.R., Perez A.T., Raanan M.G., Spanheimer R.G. (2013). Effects of pioglitazone on bone in postmenopausal women with impaired fasting glucose or impaired glucose tolerance: A randomized, double-blind, placebo-controlled study. J. Clin. Endocrinol. Metab..

[18] Derosa G., Tinelli C., Maffioli P. (2009). Effects of pioglitazone and rosiglitazone combined with metformin on body weight in people with diabetes. Diabetes Obes. Metab..

[19] Ruano G., Bernene J., Windemuth A., Bower B., Wencker D., Seip R.L., Kocherla M., Holford T.R., Petit W.A., Hanks S. (2009). Physiogenomic comparison of edema and BMI in patients receiving rosiglitazone or pioglitazone. Clin. Chim. Acta..

[20] Shah P., Mudaliar S. (2010). Pioglitazone: Side effect and safety profile. Expert Opin. Drug Saf..

[21] Choudhury H., Pandey M., Hua C.K., Mun C.S., Jing J.K., Kong L., Ern L.Y., Ashraf N.A., Kit S.W., Yee T.S. (2018). An update on natural compounds in the remedy of diabetes mellitus: A systematic review. J. Tradit. Complement. Med..

[22] Fukai M., Tsukada M., Miki K., Suzuki T., Sugita T., Kinoshita K., Takahashi K., Shiro M., Koyama K. (2012). Hypoxylonols C–F, Benzo [j] fluoranthenes from Hypoxylon truncatum. J. Nat. Prod..

[23] Hwang B.S., Lee D., Choi P., Kim K.S., Choi S.-J., Song B.G., Kim T., Song J.H., Kang K.S., Ham J. (2018). Renoprotective effects of hypoxylonol C and F isolated from hypoxylon truncatum against cisplatin-induced cytotoxicity in LLC-PK1 cells. Int. J. Mol. Sci..

[24] Stadler M., Wollweber H., Muhlbauer A., Henkel T., Asakawa Y., Hashimoto T., Ju Y.-M., Rogers J.D., Wetzstein H.-G., Thchy H.-V. (2001). Secondary metabolite profiles, genetic fingerprints and taxonomy of Daldinia and allies. Mycotaxon.

[25] Lee H., Kim J., Park J.Y., Kang K.S., Park J.H., Hwang G.S. (2017). Processed *Panax ginseng*, sun ginseng, inhibits the differentiation and proliferation of 3T3-L1 preadipocytes and fat accumulation in Caenorhabditis elegans. J. Ginseng Res..

[26] Guon T., Chung H.S. (2017). Induction of apoptosis with *Moringa oleifera* fruits in HCT116 human colon cancer cells via intrinsic pathway. Nat. Prod. Sci..

[27] Shim S., Lee S., Kim M., Lee J.W., Hwang B.Y., Lee M. (2017). Falcarindiol from *Angelica koreana* down-regulated IL-8 and up-regulated IL-10 in colon epithelial cells. Nat. Prod. Sci..

[28] Park R., Lee K.I., Kim H., Jang M., Ha T.K.Q., Oh W.K., Park J. (2017). Reserpine treatment activates AMP activated protein kinase (AMPK). Nat. Prod. Sci..

[29] Wilcox G. (2005). Insulin and insulin resistance. Clin. Biochem. Rev..

[30] Rhodes C.J. (2005). Type 2 diabetes-a matter of beta-cell life and death?. Science.

[31] Fujimoto K., Polonsky K.S. (2009). Pdx1 and other factors that regulate pancreatic β-cell survival. Diabetes Obes. Metab..

[32] Otani K., Kulkarni R.N., Baldwin A.C., Krutzfeldt J., Ueki K., Stoffel M., Kahn C.R., Polonsky K.S. (2004). Reduced β-cell mass and altered glucose sensing impair insulin-secretory function in βIRKO mice. Am. J. Physiol. Endocrinol. Metab..

[33] Fang D., Huang Z., Guan H., Liu J., Yao B., Xiao H., Li Y. (2012). The Akt/FoxO1/p27 pathway mediates the proliferative action of liraglutide in β cells. Mol. Med. Rep..

[34] Kittl M., Beyreis M., Tumurkhuu M., Furst J., Helm K., Pitschmann A., Gaisberger M., Glasl S., Ritter M., Jakab M. (2016). Quercetin stimulates insulin secretion and reduces the viability of rat INS-1 beta-cells. Cell. Physiol. Biochem..

[35] Chen L., Zhao Y., Zheng D., Ju S., Shen Y., Guo L. (2013). Orexin a affects INS-1 rat insulinoma cell proliferation via orexin receptor 1 and the AKT signaling pathway. Int. J. Endocrinol..

[36] Liu J.H., Guo L.X., Yin F., Zhang Y.L., Liu Z.X., Wang Y.W. (2013). Geniposide regulates glucose-stimulated insulin secretion possibly through controlling glucose metabolism in INS-1 cells. PLoS ONE.

[37] Liu S., Li X., Wu Y., Duan R., Zhang J., Du F., Zhang Q., Li Y., Li N. (2017). Effects of vaspin on pancreatic β cell secretion via PI3K/Akt and NF-κB signaling pathways. PLoS ONE.

[38] Gupta D., Leahy A.A., Monga N., Peshavaria M., Jetton T.L., Leahy J.L. (2013). Peroxisome proliferator-activated receptor γ (PPARγ) and its target genes are downstream effectors of FoxO1 protein in islet β-cells: Mechanism of β-cell compensation and failure. J. Biol. Chem..

