# Supplementary material for: Hypoxylonol F Isolated from Annulohypoxylon annulatum Improves Insulin Secretion by Regulating Pancreatic β-cell Metabolism
Source: Biomolecules. 2019 Aug 2;9(8):335. doi: 10.3390/biom9080335 (PMC6723394; doi:10.3390/biom9080335)
Supplement: Supplementary file 1 [file biomolecules-09-00335-s001.pdf]

Supplementary Materials

Hypoxylonol F isolated from *Annulohypoxylon annulatum* improves insulin secretion by regulating pancreatic β-cell metabolism

Dahae Lee[1,2,†], Buyng Su Hwang[3,†], Pilju Choi[4,†], Taejung Kim[4], Youngseok Kim[4], Bong Geun Song[4], Noriko Yamabe[2], Gwi Seo Hwang[2], Ki Sung Kang[2,*] and Jungyeob Ham[3,5,*]

1 School of Pharmacy, Sungkyunkwan University, Suwon 16419, Korea; pjsldh@naver.com (D.L.)

2 College of Korean Medicine, Gachon University, Seongnam 13120, Korea; norikoy@gachon.ac.kr (N.Y.); seoul@gachon.ac.kr (G.S.H.)

3 Freshwater Bioresources Utilization Bureau, Nakdonggang National Institute of Biological Resources, Sangju 37242, Korea; hwang1531@nnibr.re.kr (B.S.H.)

4 Natural Products Research Institute, Korea Institute of Science and Technology, Gangneung 25451, Korea; 112074@naver.com (P.C.); kgsing@kist.re.kr (T.K.); 114036@kist.re.kr (Y.K.); t16374@kist.re.kr (B.G.S.)

5 Division of Bio-Medical Science and Technology, University of Science and Technology, Daejeon 34114, Korea

* Correspondence: kkang@gachon.ac.kr; Tel.: +82-31-750-5402 (K.S.K.) and ham0606@kist.re.kr; Tel.: +82-33-650-3502 (J.H.)

Contents

Characterization data....................2

Scheme S1. Fractionation schemes of *Annulohypoxylon annulatum*....................3

Figure S1. HPLC chromatograms of *Annulohypoxylon annulatum* extract (A), Hypoxylonol C (B), Hypoxylonol F (C), BNT (D)....................4

Figure S2. ^{1}H NMR NMR spectrum of hypoxylonol C (**1**)....................5

Figure S3. ^{13}C NMR NMR spectrum of hypoxylonol C (**1**)....................5

Figure S4. HRMS of hypoxylonol C (**1**)....................6

Figure S5. ^{1}H NMR NMR spectrum of hypoxylonol F (**2**)....................7

Figure S6. ^{13}C NMR NMR spectrum of hypoxylonol F (**2**)....................7

Figure S7. HRMS of hypoxylonol F (**2**)....................8

Figure S8. ^{1}H NMR NMR spectrum of BNT (**3**)....................9

Figure S9. ^{13}C NMR NMR spectrum of BNT (**3**)....................9

Figure S10. HRMS of BNT (**3**)....................10

Figure S11. Effect of hypoxylonol F (2) on the protein expression levels of PPARγ, P-IRS-2, IRS-2, P-PI3K, PI3K, P-Akt (Ser473), Akt, and PDX-1 in INS-1 cells....................11

Characterization data

Hypoxylonol C (1).
Yellow amorphous powder. ^{1}H NMR (400 MHz, acetone-d_6) δ 12.61 (s, 1H, OH-9), 8.65 (s, 1H, OH-4), 7.56 (t, 1H, J = 8.0 Hz, H-11), 7.49 (dd, 1H, J = 8.0, 1.2 Hz, H-12), 7.28 (d, 1H, J = 8.0 Hz, H-6), 6.84 (dd, 1H, J = 8.0, 1.2 Hz, H-10), 6.68 (d, 1H, J = 8.0 Hz, H-5), 5.59 (dd, 1H, J = 8.5, 4.2 Hz, H-1), 5.48 (m, 1H, H-3), 5.14 (brd, 1H, OH-3), 4.28 (s, 1H, OH-1), 4.11 (dd, 1H, J = 13.8, 5.5 Hz, H-6b), 3.38 (dd, 1H, J = 16.5, 5.6 Hz, H-7), 2.47 (dt, 1H, J = 13.0, 4.3 Hz, H-2), 2.31 (dd, 1H, J = 16.4, 14.0 Hz, H-7), 2.14 (ddd, 1H, J = 13.0, 8.5, 3.2 Hz, H-2); ^{13}C NMR (100 MHz, acetone-d_6) δ 206.2 (C-8), 163.8 (C-9), 155.9 (C-4), 144.3 (C-12d), 139.6 (C-12a), 138.3 (C-12c), 137.9 (C-12b), 137.7 (C-11), 136.6 (C-6a), 123.7 (C-6), 120.8 (C-3a), 119.0 (C-12), 117.2 (C-10), 115.7 (C-8a), 114.4 (C-5), 65.5 (C-3), 62.8 (C-1), 49.9 (C-6b), 43.7 (C-7), 42.4 (C-2); HRFABMS m/z 335.0925 $[M-H]^{-}$ (calcd for $C_{20}H_{15}O_5$, 335.0919).

Hypoxylonol F (2).
Yellow amorphous powder. ^{1}H NMR (400 MHz, acetone-d_6) δ 12.60 (s, 1H, OH-9), 7.53 (t, 1H, J = 8.0 Hz, H-11), 7.48 (dd, 1H, J = 8.0, 1.2 Hz, H-12), 7.29 (d, 1H, J = 8.0 Hz, H-6), 6.82 (dd, 1H, J = 8.0, 1.2 Hz, H-10), 6.69 (d, 1H, J = 8.0 Hz, H-5), 5.38 (d, 1H, J = 8.5 Hz, H-1), 5.11 (d, 1H, J = 8.5 Hz, H-3), 4.09 (dddd, 1H, J = 13.8, 5.4, 23.9, 0.7 Hz, H-6b), 3.39 (dd, 1H, J = 16.4, 5.5 Hz, H-7), 2.47 (dt, 1H, J = 12.6, 4.3 Hz, H-2), 2.34 (dd, 1H, J = 16.3, 13.8 Hz, H-7), 2.24 (dt, 1H, J = 12.6, 8.5 Hz, H-2); ^{13}C NMR (100 MHz, acetone-d_6) δ 206.3 (C-8), 163.6 (C-9), 155.7 (C-4), 144.2 (C-12d), 139.4 (C-12a), 139.0 (C-12c), 137.5 (C-12b), 137.1 (C-11), 136.6 (C-6a), 123.8 (C-6), 121.0 (C-3a), 120.9 (C-12), 117.0 (C-10), 115.8 (C-8a), 114.6 (C-5), 67.0 (C-3), 65.2 (C-1), 50.1 (C-6b), 43.7 (C-7), 43.3 (C-2); HRFABMS m/z 337.1074 $[M+H]^{+}$ (calcd for $C_{20}H_{17}O_5$, 337.1076).

4,5,4',5'-Tetrahydroxy-1,1'-binaphthyl, BNT (3).
Yellow amorphous powder. ^{1}H NMR (400 MHz, acetone-d_6) δ 11.04 (br d, 4H, OH-4, 5), 7.16 (d, 2H, J = 8.0 Hz, H-7), 7.07 (t, 2H, J = 8.0 Hz, H-2), 6.83 (d, 2H, J = 8.0 Hz, H-3), 6.72 (dd, 2H, J = 8.0, 1.3 Hz, H-6), 6.59 (d, 2H, J = 8.0, 1.3 Hz, H-8); ^{13}C NMR (100 MHz, acetone-d_6) δ 155.4 (C-5), 154.8 (C-4), 137.3 (C-8a), 131.2 (C-1), 129.8 (C-7), 127.6 (C-2), 119.2 (C-8), 115.8 (C-4a), 109.7 (C-6), 109.3 (C-3); HRFABMS m/z 317.0778 $[M-H]^{+}$ (calcd for $C_{20}H_{13}O_4$, 317.0773)

Scheme S1. Fractionation schemes of *Annulohypoxylon annulatum*

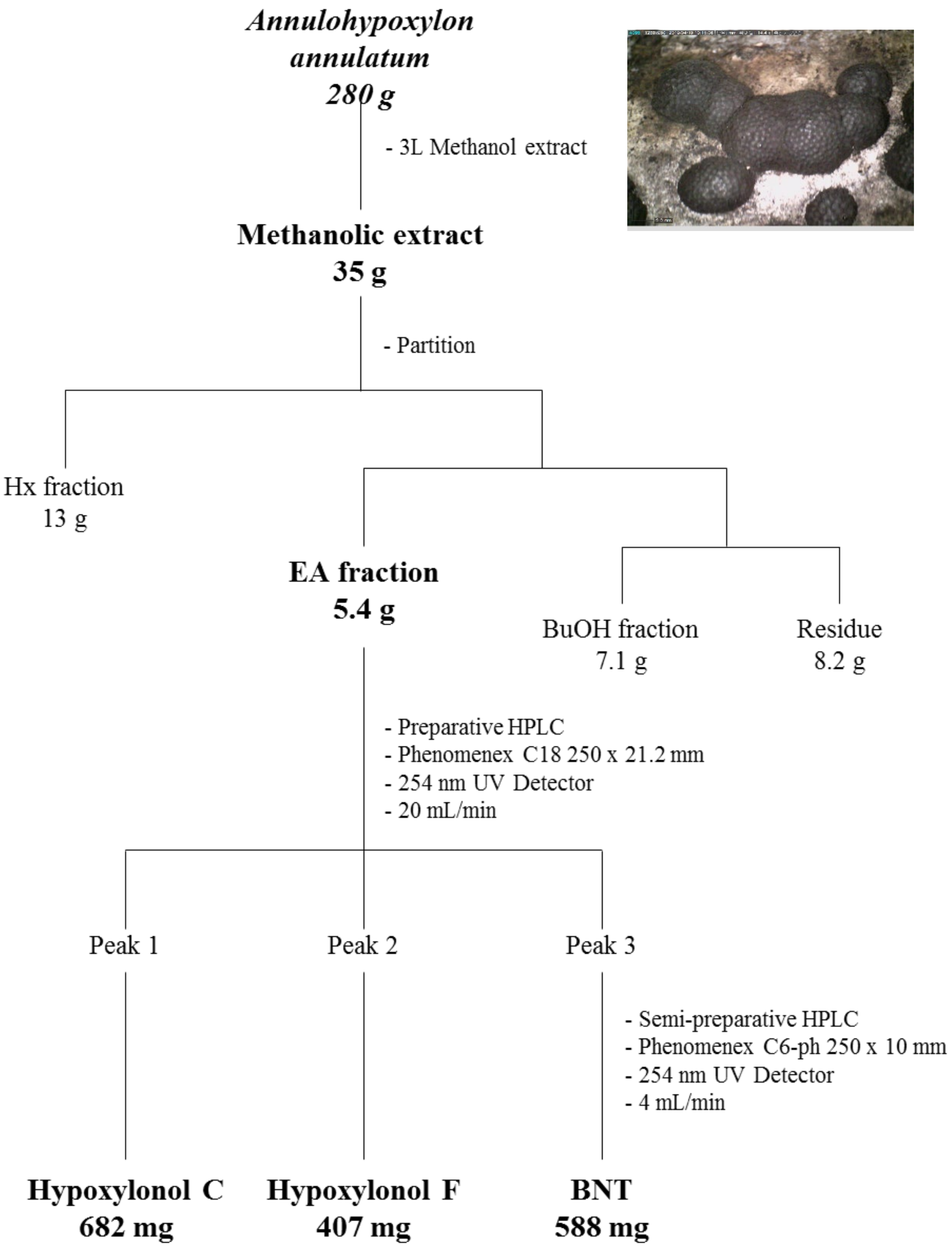

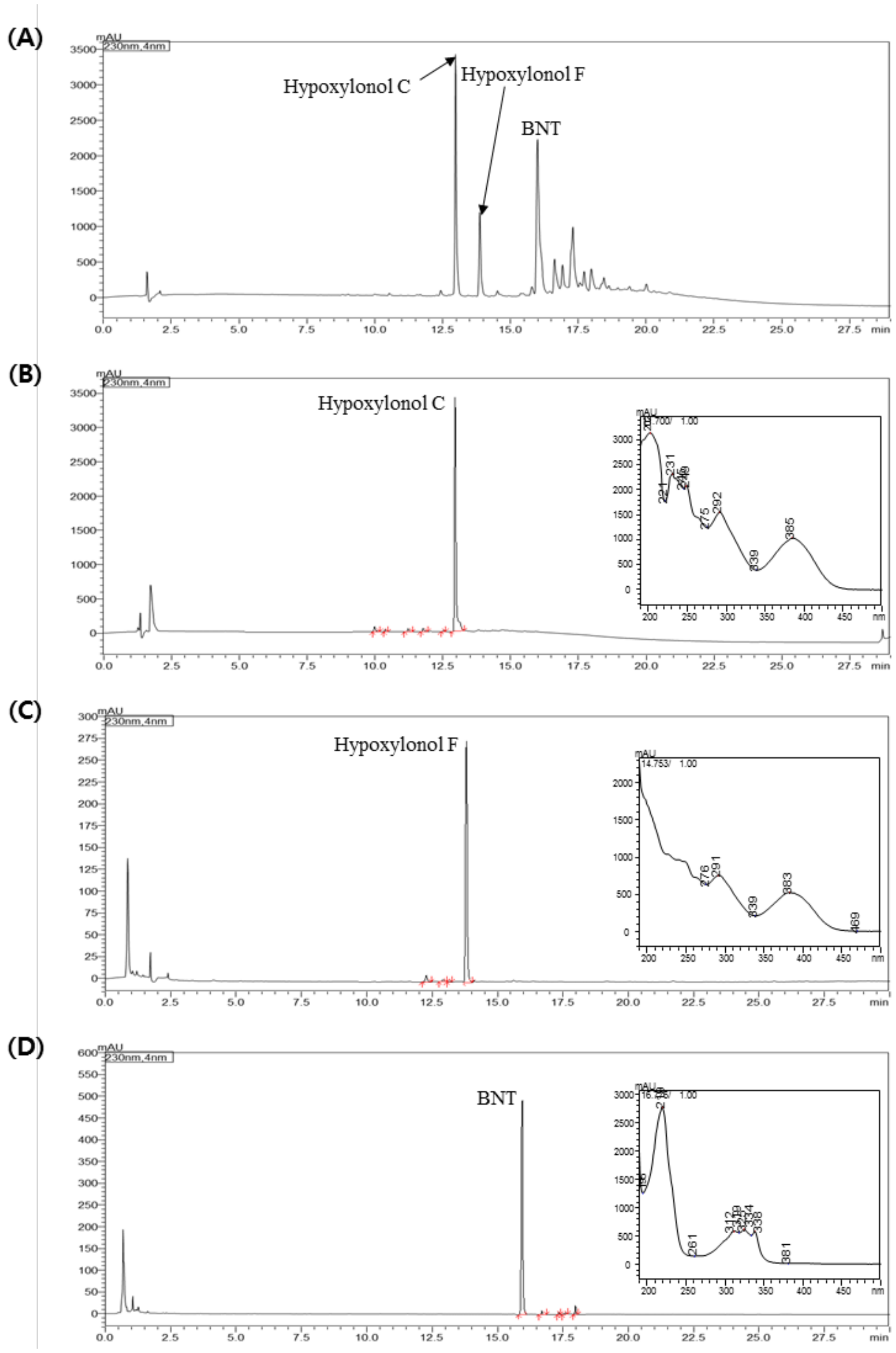

Figure S1. HPLC chromatograms of *Annulohypoxylon annulatum* extract (A), Hypoxylonol C (B), Hypoxylonol F (C), BNT (D).

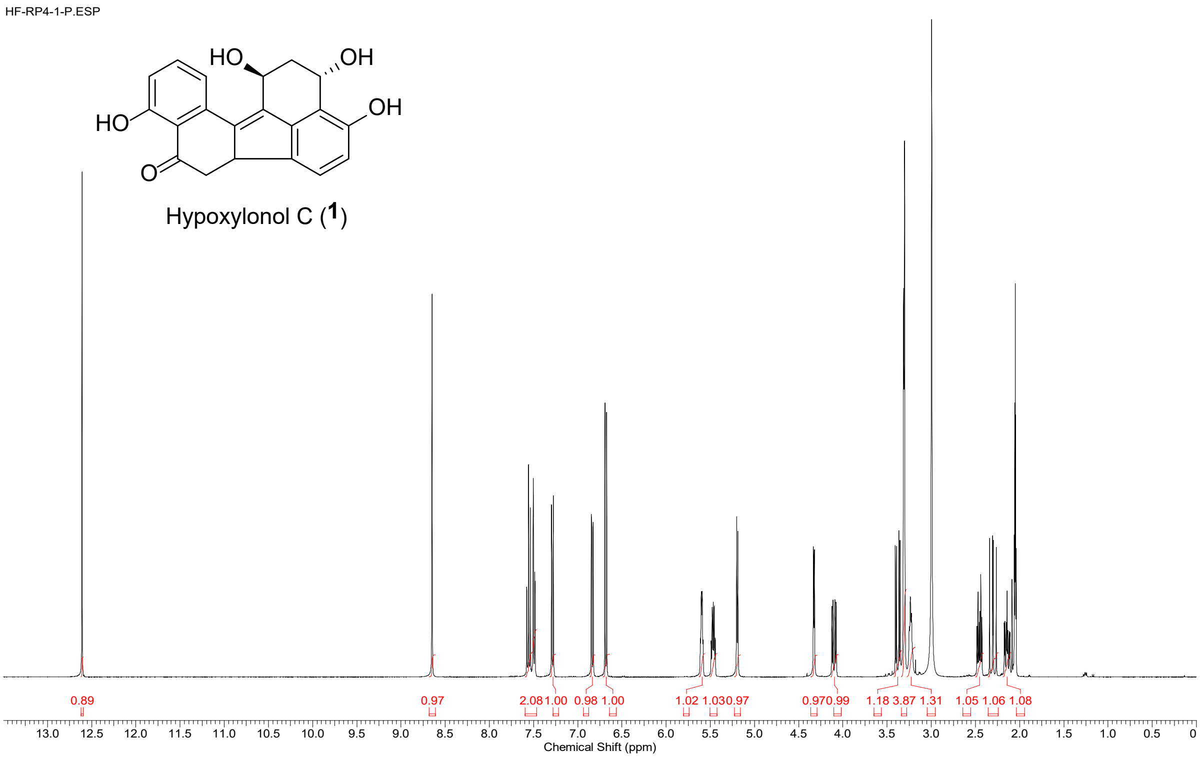

Figure S2. ^{1}H NMR NMR spectrum of hypoxylonol C (**1**)

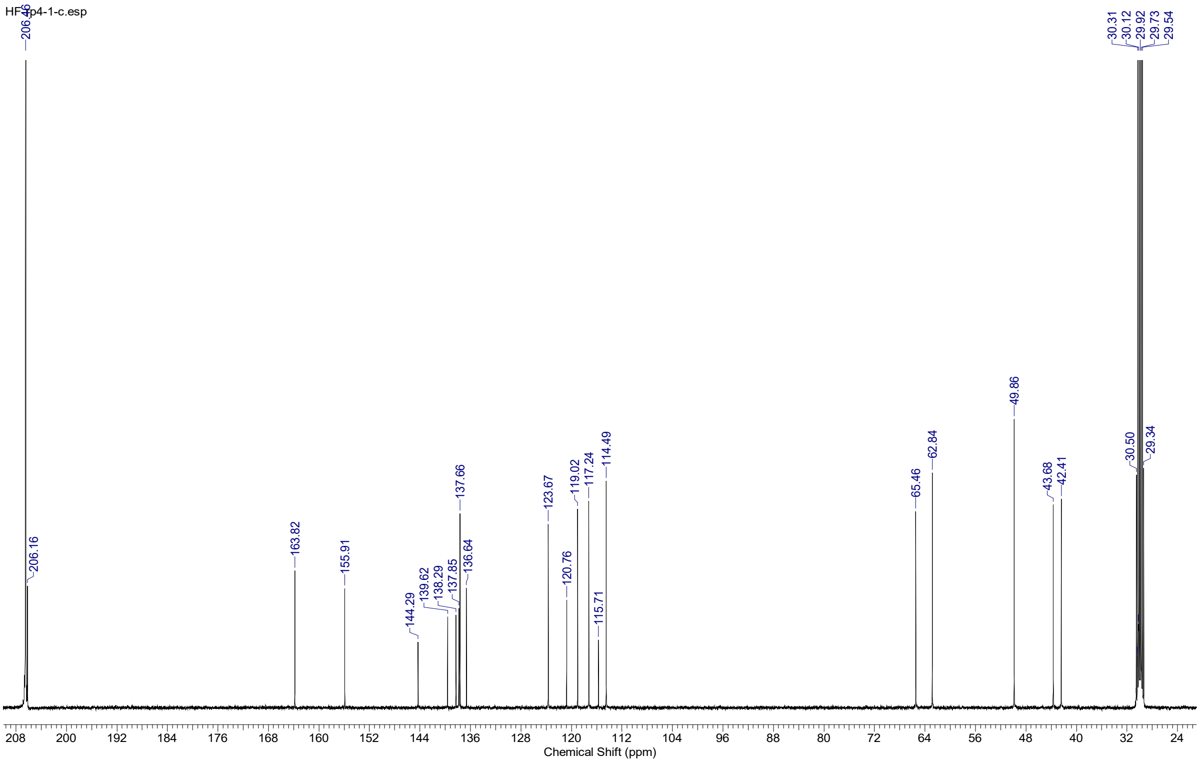

Figure S3. ^{13}C NMR NMR spectrum of hypoxylonol C (**1**)

[Mass Spectrum]
Data : KIST_known_001
Sample : -
Note : -
Inlet : Direct   Ion Mode : EI-

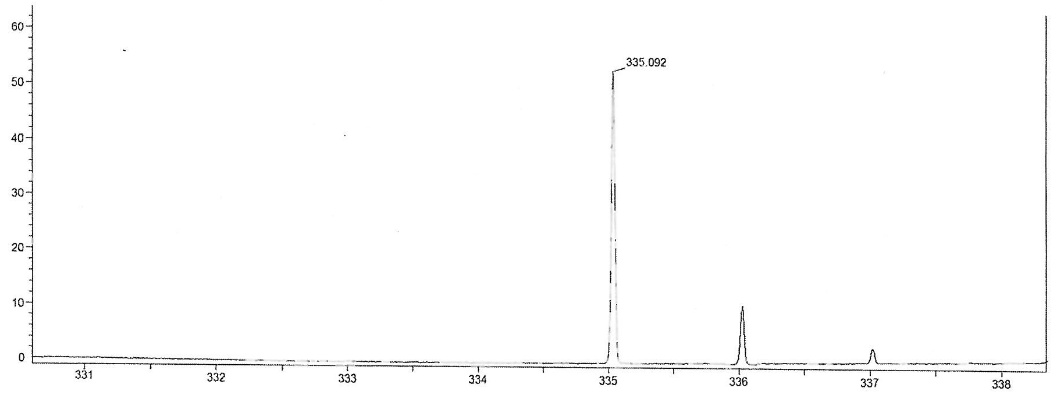

[Theoretical Ion Distribution]

Molecular Formula : C20 H15 O5 [M-H]-

(m/z 335.0919, MW 335.3341)

Base Peak : 335.0919, Averaged MW : 335.3145(a), 335.3316(w)

m/z	INT	
335.0919	100.0000	**
336.0942	21.4650	*************
337.0968	2.1852	**
338.0991	0.2468	
339.1104	0.0214	

Figure S4. HRMS of hypoxylonol C (**1**)

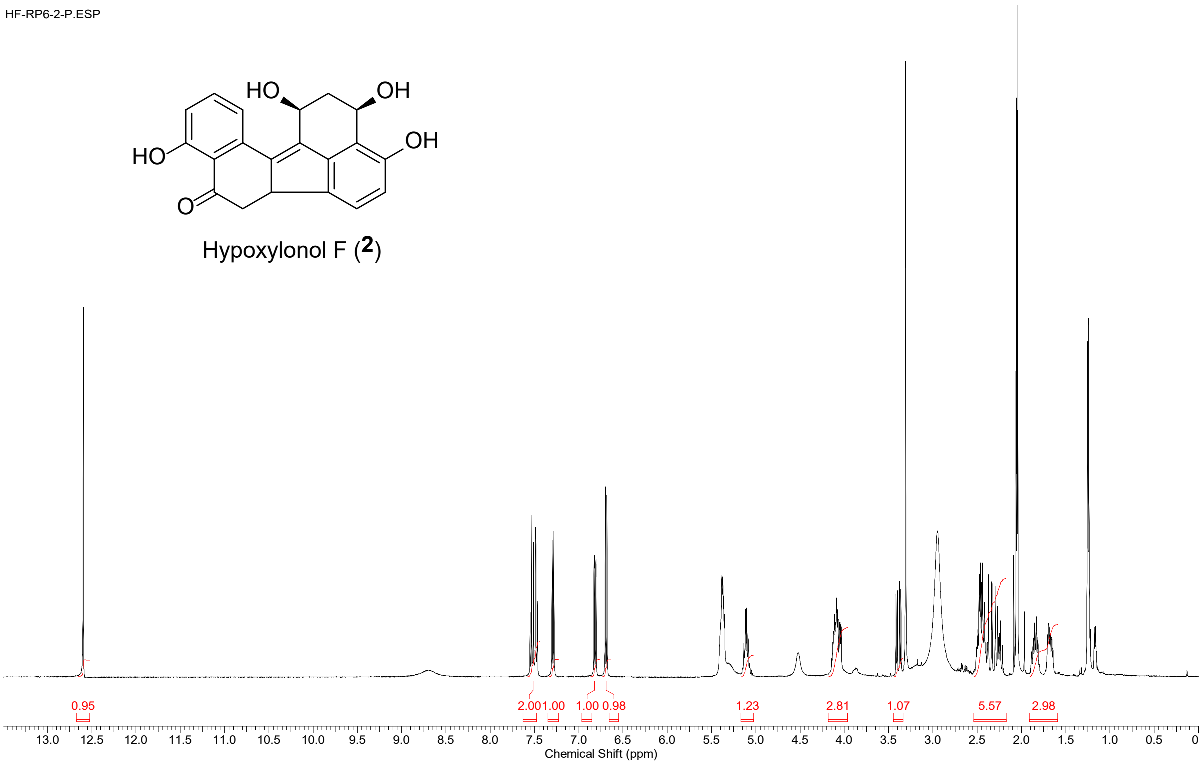

Figure S5. ^{1}H NMR NMR spectrum of hypoxylonol F (**2**)

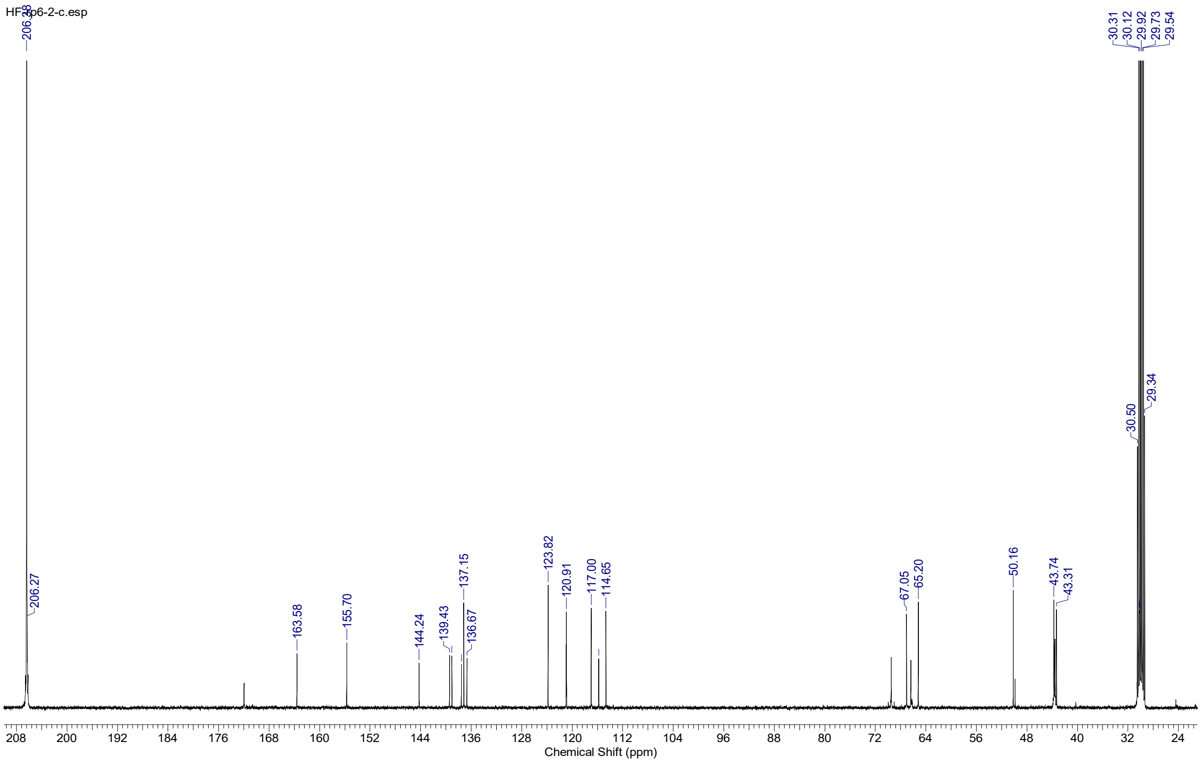

Figure S6. ^{13}C NMR NMR spectrum of hypoxylonol F (**2**)

[Mass Spectrum]
Data : KIST_known_002
Sample : -
Note : -
Inlet : Direct Ion Mode : EI+

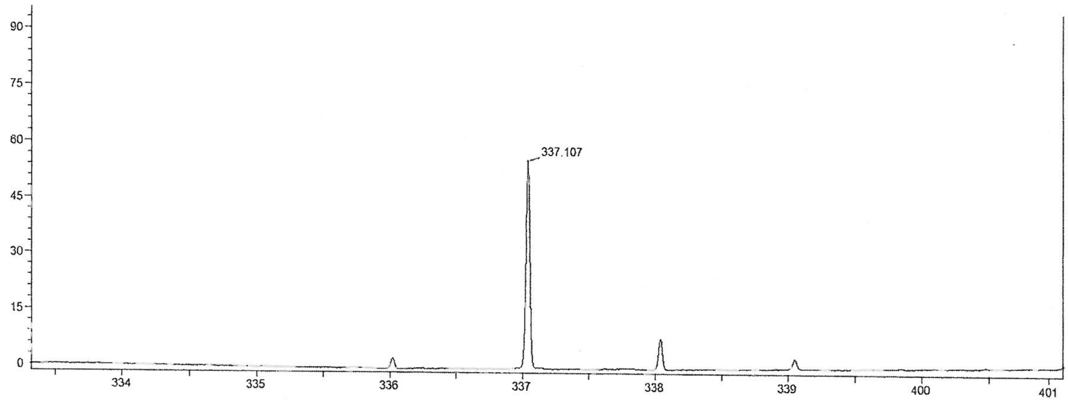

[Theoretical Ion Distribution]
Molecular Formula : C20 H17 O5 [M+H]+
(m/z 337.1074, MW 337.3511)
Base Peak : 337.1074, Averaged MW : 337.3445(a), 337.3496(w)

m/z	INT
337.1074	100.0000
338.1081	21.9650
339.1088	2.2852
400.1094	0.1975
401.1102	0.0182

Figure S7. HRMS of hypoxylonol F (**2**)

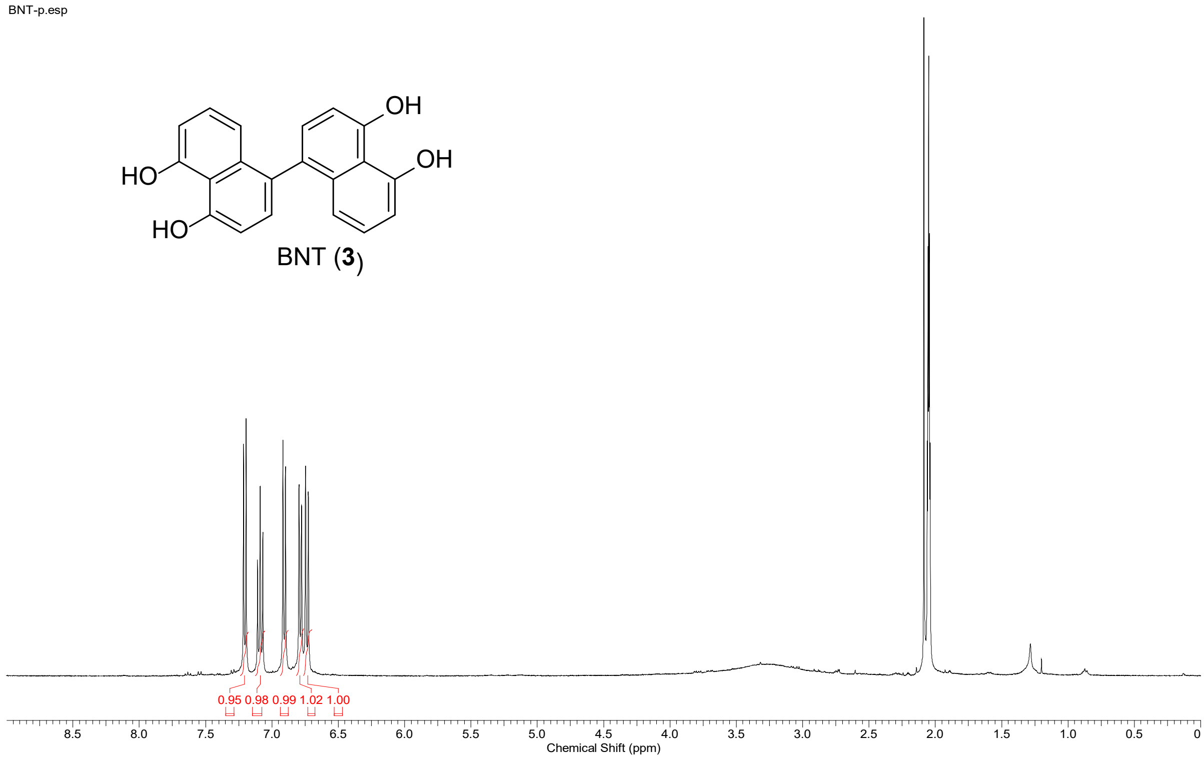

Figure S8. ^{1}H NMR NMR spectrum of BNT (**3**)

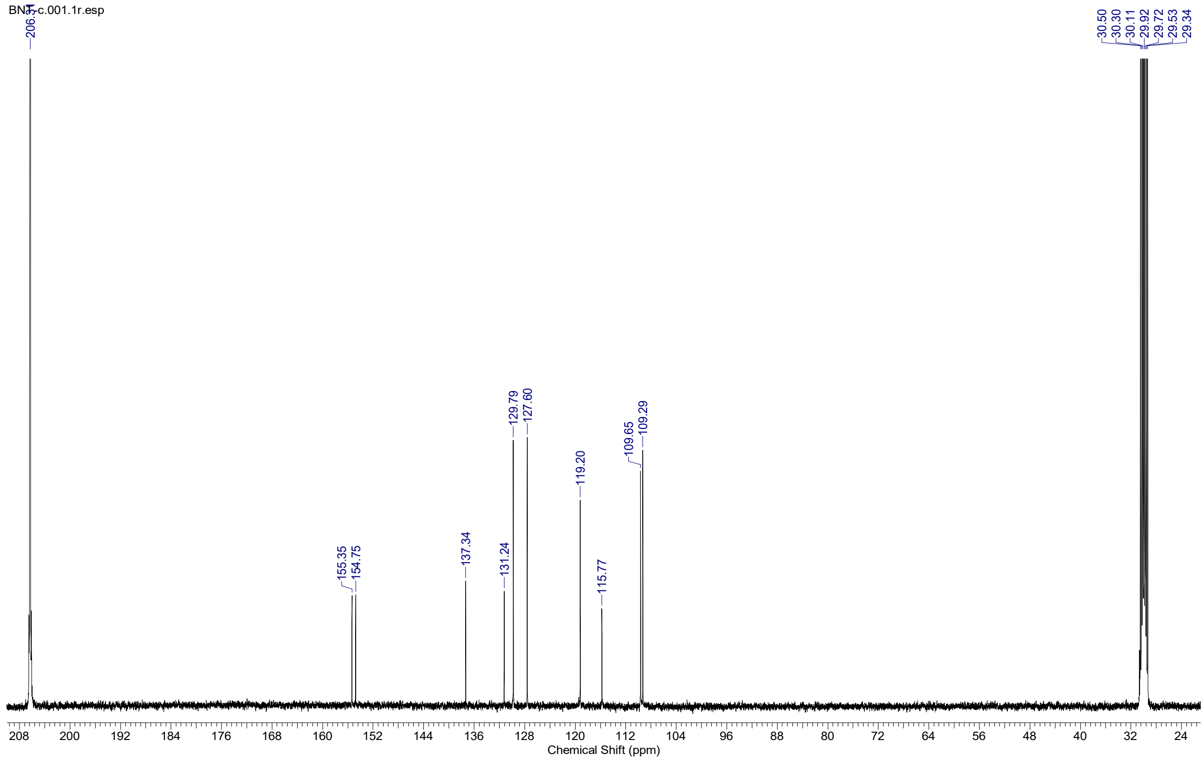

Figure S9. ^{13}C NMR NMR spectrum of BNT (**3**)

[Mass Spectrum]
Data : KIST_known_003
Sample : -
Note : -
Inlet : Direct   Ion Mode : EI+

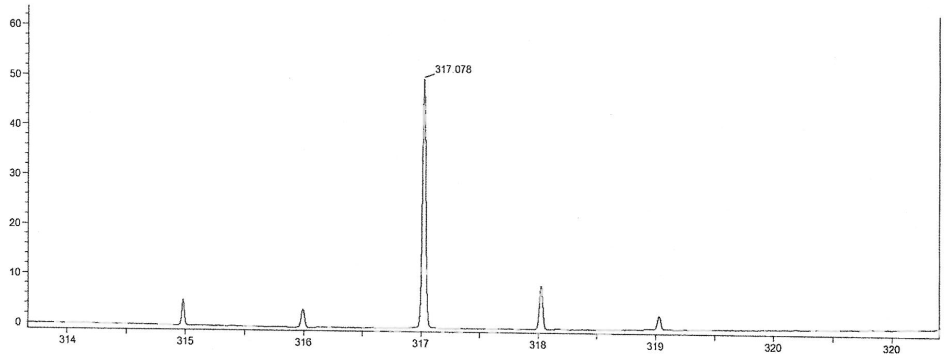

[Theoretical Ion Distribution]
Molecular Formula : C20 H13 O4 $[M+H]^+$
(m/z   317.0778,   MW   317.3199)
Base Peak :   317.0778, Averaged MW :   317.3045(a),   317.3156(w)

m/z	INT	
317.0778	100.0000	**
318.0792	20.8460	**************
319.0808	1.9912	**
320.0821	0.2237	
321.0834	0.0197	

Figure S10. HRMS of BNT (**3**)

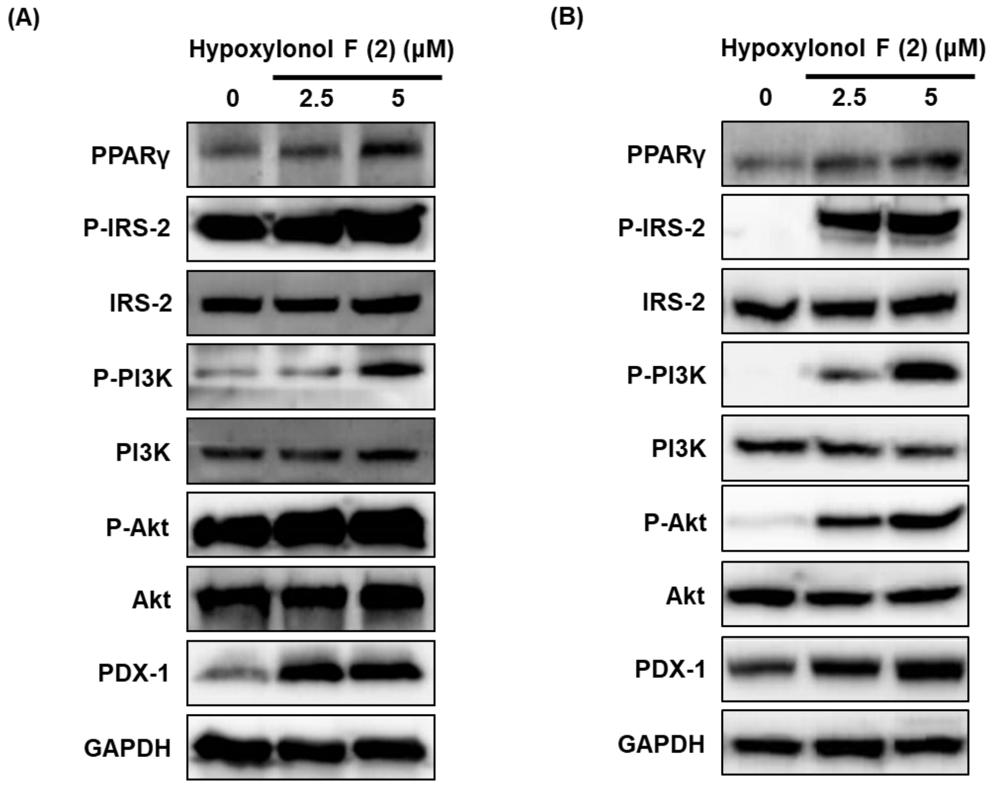

Figure S11. Effect of hypoxylonol F (**2**) on the protein expression levels of PPARγ, P-IRS-2, IRS-2, P-PI3K, PI3K, P-Akt (Ser473), Akt, and PDX-1 in INS-1 cells.
